# Rituximab in refractory Vogt–Koyanagi–Harada disease

**DOI:** 10.1007/s12348-011-0027-9

**Published:** 2011-07-09

**Authors:** Rosa Dolz-Marco, Roberto Gallego-Pinazo, Manuel Díaz-Llopis

**Affiliations:** 1 Department of Ophthalmology, University and Polytechnic Hospital La Fe, Valencia, Spain; 2 Faculty of Medicine, University of Valencia, Valencia, Spain

**Keywords:** Chronic VKH, Rituximab, Immunommodulators

## Abstract

**Introduction:**

Vogt–Koyanagi–Harada (VKH) prognosis depends on early recognition and treatment; chronic disease may be developed when either delayed or inadequate treatment is performed, whereas other cases despite correct treatment are refractory to different drugs and also become chronic. We report a case of refractory VKH controlled with rituximab treatment.

**Case report:**

A 41-year-old female with painful visual loss and headache was examined. (VA 0.4 in RE and hand movements (HM) in LE). Retinal examination demonstrated multiple serous retinal detachments in both eyes. High-dose oral steroids were started, followed by progressive tapering of prednisone. New acute anterior and posterior relapses were achieved, and other immunommodulators were progressively added—new high-dose steroid treatment, adalimumab, cyclosporine, and methotrexate—but patient had new anterior and posterior recurrences associated with tinnitus and headache. Thus, an infusion of 1 g of rituximab was administered after 15 months follow-up; the VA was 0.2 in RE and counting fingers in LE. Three additional doses of 1 g each were administered 1, 6, and 16 months later. We have achieved a final VA after 34 months follow-up of 0.2 in RE and HM in LE, with definitive control of inflammation, without acute relapses since rituximab was administered.

**Conclusion:**

After searching PubMed/Medline, this is the first report of VKH disease treated with rituximab. Additional studies are warranted to confirm the efficacy of this new approach for inflammatory control in refractory cases of VKH disease.

## Introduction

Vogt–Koyanagi–Harada (VKH) disease is a T-cell mediated autoimmune disorder with an acute phase of severe bilateral panuveitis and exudative retinal detachment. Patients adequately treated with high-dose systemic corticosteroids at the onset of the disease frequently have rapid resolution of inflammation with good visual outcome [1]. However, more than half of these patients, particularly those who receive either delayed or inadequate treatment, may develop chronic disease with consequent poor visual prognosis due to cataract, glaucoma, subretinal fibrosis, choroidal neovascularization, or choroidal atrophy [2]. These complications are accompanied by alterations in the retinal pigment epithelium in the form of hypo or hyperplasia or granular changes leading to a sunset glow appearance associated in some cases with Dalen-Fuchs nodules [3]. Thus, to prevent recurrences or to control these when chronic disease is developed, in non-responsive patients or when corticosteroid side effects are not well tolerated, other immunomodulatory agents may be needed (corticosteroid-saving agents) [4].

Rituximab is a human–murine chimeric monoclonal antibody targeting CD20, a transmembrane protein present on immature and mature B cells. It was first approved for the treatment of non-Hodgkin's B-cell lymphoma, and it is very effective for depleting both normal and malignant CD20 B cells in vivo. There are only a few publications about the efficacy and tolerability of rituximab treatment in patients with autoimmune ocular inflammatory diseases. It has been used in cases of refractory endogenous anterior uveitis and Wegener's granulomatosis associated with peripheral ulcerative keratitis [5, 6]. Other uses in ophthalmology have been reported, such as corticosteroid-resistant thyroid-associated ophthalmopathy or in patients with juvenile idiopathic arthritis-associated uveitis [7, 8].

In an automatic search in PubMed/Medline, we did not find any report about the use of rituximab for either acute or chronic VKH.

## Case report

A 41-year-old Caucasian female from Argentina attended to our department complaining of bilateral painful visual loss, associated with headache and tinnitus.

**Fig. 1:**
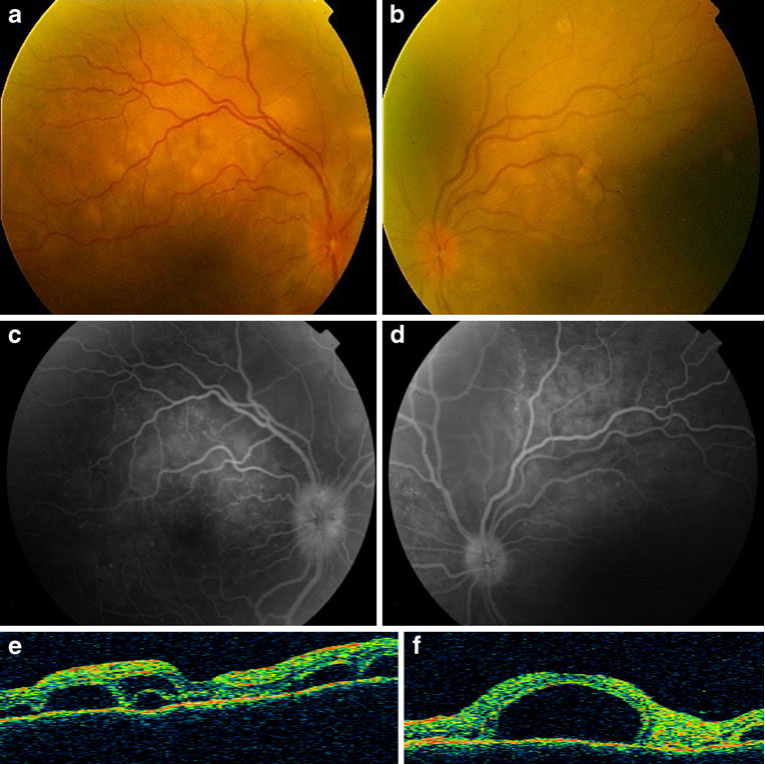
**a, b.** Color fundus images of right and left eye showed disc edema and multifocal serous retinal detachment with foveal involvement. **c, d.** Fluorescein angiography photographs of both eyes revealed characteristic pinpoint hyperfluorescence in intermediate time. **e, f.** Time domain optical coherence tomography cross-sectional horizontal image showed typical multilobulated retinal serous detachment in both eyes

Her best corrected visual acuity (BCVA) was 0.4 in her right eye (RE) and counting fingers in her left eye (LE). The anterior chamber showed perikeratic hyperemia, moderate anterior chamber flare, and 1+ Tyndall phenomenon in both eyes. Fundus examination evidenced bilateral disc edema and multifocal serous retinal detachment with foveal involvement. Fluorescein angiography revealed late pinpoint hyperfluorescence. Optical coherence tomography showed multilobulated retinal serous detachment. Lumbar puncture revealed pleocytosis with polymorphonuclear cells (Fig. 1).

**Fig. 2:**
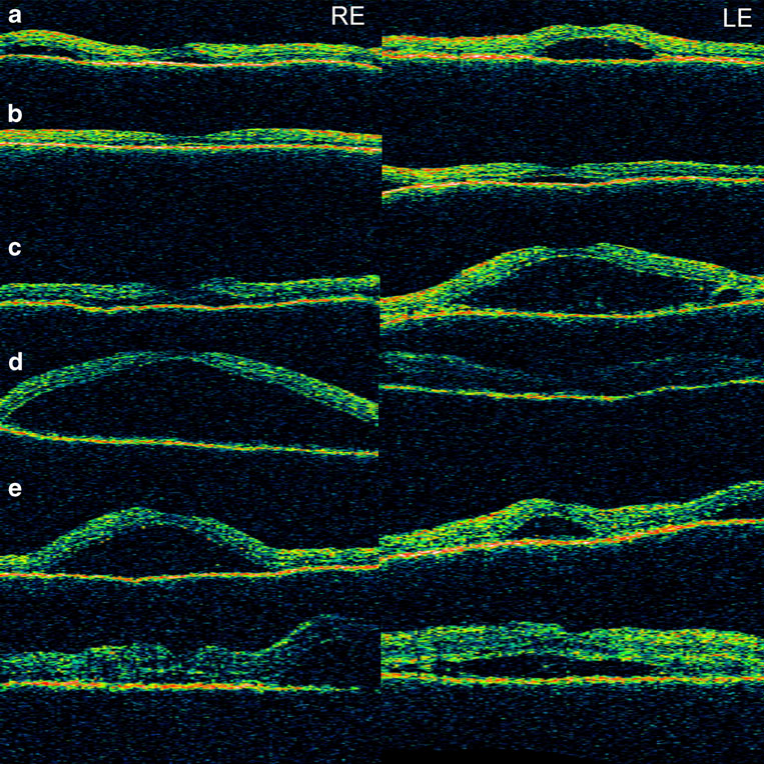
Optical coherence tomography cross-sectional horizontal images of right and left eye (*RE* and *LE*, respectively); **a** (2 days follow-up) showed response to high-dose corticosteroids in both eyes with decrease of neuroretinal detachment that is more evident after 2 weeks of follow-up in **b**. **c** (3 months follow-up) revealed first recurrence of macular detachment in left eye first and later in right eye (**d**, 4 months follow-up) treated with a new high-dose oral corticosteroid therapy associated with bilateral intravitreal injection of ranibizumab and dexamethasone and adalimumab every 15 days. **f** (5 months follow-up) showed reappearance of retinal detachment, so we added treatment with cyclosporine (100 mg per day) and methotrexate (17.5 mg weekly). **g** (7 months follow-up) revealed macular detachment, and examination showed recurrence of anterior and posterior inflammation with tinnitus and headache; thus, we started a first infusion of 1 g of rituximab (15 months after onset of symptoms)

High-dose oral steroid treatment was started (1 g daily of methylprednisolone for 3 days), followed by gradual tapering of oral prednisone, achieving a significant improvement of BCVA (0.6 and 0.4). Three months later, a new episode of posterior uveitis was evidenced, decreasing VA to 0.05 in her RE and 0.02 in her LE. Due to this exacerbation, treatment with new high-dose oral corticosteroid dose, bilateral intravitreal injection of ranibizumab and dexamethasone and adalimumab (Humira®) every 15 days was performed. However, a new relapse of anterior and posterior inflammation occurred, so cyclosporine (200 mg daily) and methotrexate (17.5 mg weekly) were added to the treatment (Fig. 2). Despite this combination therapy, further constant recurrences of anterior chamber inflammation, retinal fluid relapses, tinnitus, and headache were registered; thus, infusion of 1 g of rituximab was performed (15 months after onset of symptoms). BCVA at this moment was 0.2 in her RE and hands motion (HM) in her LE. Three additional infusions of 1 g each were administered, 1, 6, and 16 months later.

**Fig. 3:**
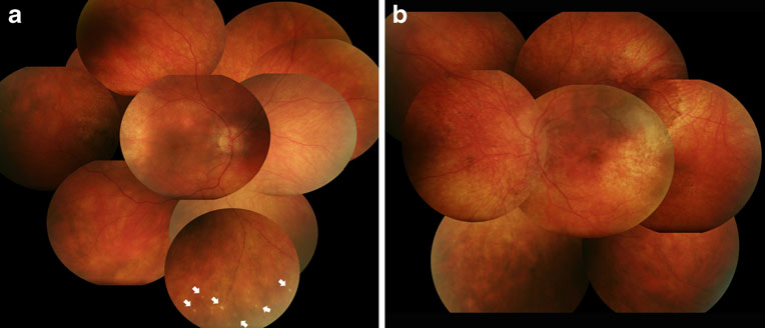
**a**, **b**. Color fundus photography of right and left eye, respectively, after 30 months follow-up showed typical diffuse athrophy of retinal pigment epithelium leading to a characteristic image of sunset-glow fundus. **a** (*arrows*) revealed the presence of Dalen-Fuchs nodules

We obtained an optimal response with definitive control of inflammation, without further relapses, since rituximab was administered with no more decrease of BCVA at 34 months follow-up (Fig. 3).

## Discussion

VKH prognosis depends on early recognition and aggressive suppression of inflammation. The use of high-dose steroids and other immunosuppressant agents has improved significantly the visual outcome [9]; however, VKH may be a disease difficult to treat with chronic relapses and may require the use of other immunosuppressive drugs like TNF blockers (infliximab or adalimumab) [10, 11] or different combination therapy [4]. Intravitreal treatments with steroids and vascular endothelial growth factor inhibitors have been also reported as adjuvant possibilities with variable results [12–14].

It may surprise the effectiveness of treatment with an anti CD-20 monoclonal antibody in a T-cell-mediated disease; however, changes in B and T lymphocytes after rituximab treatment in multiple sclerosis have been analyzed. Unexpectedly, after B-cell depletion, a decline in cerebrospinal fluid and blood T cells also occurred [15].

In this case, we started treatment with high-dose steroids, but it was insufficient to control the disease. Patient had an inadequate response to conventional immunosuppressive treatment, such as methotrexate, cyclosporine, and TNF blockers with constant recurrences. Consequently, we initiated rituximab therapy with an optimal control of inflammation. We achieved preservation of BCVA in RE, without relapsing inflammatory episodes, since we have started rituximab treatment.

## Conclusion

To our knowledge, this is the first report of VKH disease treated with rituximab. Although experience in treatment of inflammatory eye disease with rituximab is reduced, this therapy might be considered a therapeutic alternative for treatment of patients with autoimmune ocular diseases, particularly those who have not previously responded to TNF blockers. Additional studies are warranted to confirm the efficacy of this new approach for inflammatory control in refractory cases of VKH disease.
